# Long-term delivery of brain-derived neurotrophic factor (BDNF) from nanoporous silica nanoparticles improves the survival of spiral ganglion neurons in vitro

**DOI:** 10.1371/journal.pone.0194778

**Published:** 2018-03-27

**Authors:** Nadeschda Schmidt, Jennifer Schulze, Dawid P. Warwas, Nina Ehlert, Thomas Lenarz, Athanasia Warnecke, Peter Behrens

**Affiliations:** 1 Institut für Anorganische Chemie, Leibniz Universität Hannover, Hannover, Germany; 2 Cluster of Excellence Hearing4all, Hannover, Germany; 3 Department of Otorhinolaryngology, Head and Neck Surgery, Hannover Medical School, Hannover, Germany; Helsingin Yliopisto, FINLAND

## Abstract

Sensorineural hearing loss (SNHL) can be overcome by electrical stimulation of spiral ganglion neurons (SGNs) via a cochlear implant (CI). Restricted CI performance results from the spatial gap between the SGNs and the electrode, but the efficacy of CI is also limited by the degeneration of SGNs as one consequence of SHNL. In the healthy cochlea, the survival of SGNs is assured by endogenous neurotrophic support. Several applications of exogenous neurotrophic supply have been shown to reduce SGN degeneration *in vitro* and *in vivo*. In the present study, nanoporous silica nanoparticles (NPSNPs), with an approximate diameter of <100 nm, were loaded with the brain-derived neurotrophic factor (BDNF) to test their efficacy as long-term delivery system for neurotrophins. The neurotrophic factor was released constantly from the NPSNPs over a release period of 80 days when the surface of the nanoparticles had been modified with amino groups. Cell culture investigations with NIH3T3 fibroblasts attest a good general cytocompatibility of the NPSNPs. *In vitro* experiments with SGNs indicate a significantly higher survival rate of SGNs in cell cultures that contained BDNF-loaded nanoparticles compared to the control culture with unloaded NPSNPs (p<0.001). Importantly, also the amounts of BDNF released up to a time period of 39 days increased the survival rate of SGNs. Thus, NPSNPs carrying BDNF are suitable for the treatment of inner ear disease and for the protection and the support of SGNs. Their nanoscale nature and the fact that a direct contact of the nanoparticles and the SGNs is not necessary for neuroprotective effects, should allow for the facile preparation of nanocomposites, e.g., with biocompatible polymers, to install coatings on implants for the realization of implant-based growth factor delivery systems.

## Introduction

According to the World Health Organization (February 2017), 360 million people worldwide suffer from hearing loss. From genetic defects and infectious diseases to excessive noise and aging, hearing loss has many causes and presents a heavy burden for the affected individuals [1]. One common form of deafness is the sensorineural hearing loss (SNHL) caused by damage of the hair cells within the cochlea [2–4]. For almost over 30 years now, a standard therapy of patients suffering from profound or severe SNHL is the cochlear implant, a complex bionic device. Cochlear implants can replace the function of hair cells by direct electrical stimulation of primary auditory neurons (spiral ganglion neurons, SGNs) [5,6]. However, the efficacy of cochlear implants is limited by the anatomical gap between the electrode array inserted into the scala tympani and the SGNs situated in the Rosenthal´s canal leading to an unspecific stimulation of relatively large groups of neurons [7,8]. Moreover, the performance of cochlear implants strongly depends on the number of surviving neurons and their functionality, i.e., their excitability [3,9]. As many studies reported, progressive degeneration of the SGNs is one consequence of SNHL [6,10] and this was not only observed in animal models [11], but also in humans [12]. A primary cause of SGN degeneration can be the missing neurotrophic support with growth factors by sensory hair cells and cochlear-supporting cells [13–17]. Growth factors like brain-derived neurotrophic factor (BDNF) and neurotrophin-3 (NT-3) are produced by these cells and regulate neuronal survival, differentiation of neurons and axonal growth [18,19]. Therefore, neurotrophic factors are promising as therapeutic agents to inhibit or delay degenerative processes in SGNs. In addition to supporting SGN survival, neurotrophic factors have been shown to stimulate the neurite outgrowth from SGNs [20–22], which is important for an improved electro-neural interface. Due to the limitations of the cochlear implant, strategies to improve the survival and the growth of SGNs with simultaneous electrical stimulation from cochlear implants have received much interest. Despite the fact that electrical stimulation itself may induce neurotrophic signaling pathways via depolarization [6,23,24], additional treatment with neurotrophins effectively increases the survival of SGNs [25–27]. In comparison to the historical treatment of inner ear diseases with systemic therapies, one of the most common application method for exogenous local supply with neurotrophic factors are osmotic pumps [25,28–31]. But both methods have their limitations. Systemic drug delivery on the one hand is limited by restricted blood flow to the inner ear and poor penetration of the blood-cochlea barrier [2,32]. On the other hand, the neurotrophic supply with pump-based devices is finite, because the reservoir has to be refilled periodically, and the presence of a pump offers the possibility for infection of the inner ear [6].

An alternative to pump-based neurotrophin administration is a local delivery of neurotrophins performed by cell-based and gene therapy. For example, inoculation of a viral vector containing the BDNF gene leads to expression of BDNF which results in higher SGN survival [33,34]. Nevertheless, several safety concerns, like the cell toxicity caused by certain viral vectors and the non-controllable neurotrophic expression, have to be solved before considering clinical applications [6,13,35]. Thus, current research has focused on the development of neurotrophin delivery systems that provide a safe and effective neurotrophic supply for the long term, especially if they can be combined directly with the implant (implant-associated drug delivery). Promising approaches are electrode coating materials [36–38] and carrier systems like hydrogels [39–41], microspheres [42] and nanoparticles [43–46]. Even though each carrier system has advantages and disadvantages, nanoparticular systems for growth factor delivery have recently attracted increasing interest [35,44,47,48].

In the present study we describe the use of nanoporous silica nanoparticles (NPSNPs) as a special delivery platform for neurotrophic factors. BDNF is chosen as model growth factor for this application. NPSNPs offer a great potential as delivery platforms due to their advantageous properties. These properties include a high surface area, a large pore volume (up to 50%), the amenability for surface modification as well as a general biocompatibility [49–53]. Recently, a first in-human study of these particles was reported [54]. By reaction with the surface silanol groups, the surface chemistry characteristics can be adjusted by decorating with different functional groups for an envisaged immobilization of different bioactive molecules. Moreover, via such modifications, targeted delivery can be performed by anchoring specific ligands for receptor recognition [35].

The NPSNPs used in this study have already proved to be suitable for growth factor immobilization. For example, our previous studies have shown that BMP2-loaded NPSNPs supported osteogenic differentiation of human mesenchymal stem cells [53]. Moreover, current research has presented the efficacy of BDNF-loaded supraparticles (≈ 500 μm) of mesoporous silica to improve SGN survival *in vivo* [10,43]. However, these particles are considerably larger than those used here, and to the best of our knowledge, the efficacy of NPSNPs with an approximate diameter of <100 nm as carrier systems for BDNF has not been investigated so far. The group of Praetorius has already shown that silica nanoparticles with 20 nm in size are potentially safe for use in the inner ear [55]. Nanoparticles with diameters <100 nm are amenable to the incorporation into different matrices, e.g. polymers, and are therefore useful for the construction of cochlear implant-associated delivery systems, e.g. in coatings of the electrode contacts or attached to the silicone surface. For example, we have shown in a previous study that it was possible to incorporate NPSNPs into dental composite materials [56]. When a cochlear implant is placed anyway, the incorporation of a delivery system seems very appropriate. Released BDNF can then act as a neuroprotective factor and might guide neurite outgrowth towards the cochlear electrode [37,45,57].

The main purpose of the present study is to establish the general suitability of BDNF-loaded NPSNPs for the construction of cochlear implant-associated BDNF delivery systems. Thus, apart from testing the cytocompatibility of the nanoparticles with SGNs, the question whether BDNF which is released from the nanoparticles has a positive effect on the survival of SGNs *in vitro* (without the necessity for a direct contact between nanoparticles and SGNs) is most important. In view of the fact that growth factor-based engineering of cellular behavior should aim at long-term effects, a specific focus is posed on the question whether this is also true for the amounts delivered after long release periods, which is why we studied the release for 80 days.

## Experimental

### Synthesis and modification of nanoporous silica nanoparticles

All chemicals, except absolute ethanol, were obtained commercially from Sigma Aldrich (Munich, Germany) and used without further purification. Absolute ethanol was purchased from Merck (Darmstadt, Germany).

NPSNPs were prepared by adding 3.16 g cetyltrimethylammonium bromide (CTAB) and 0.23 g diethanolamine (DEA) to a solution of 75 mL ultrapure water and 13.4 mL absolute ethanol. The mixture was heated to 40°C and stirred. After 30 min, 8.56 mL tetraethoxysilane (TEOS) were added and the reaction mixture was stirred for additional 2 h. The product was centrifuged (30 min at 18000 *g*) and washed twice with water and once with ethanol. Afterwards, the particles were dried overnight at 60°C. To remove the structure-directing agent, a calcination for 5 h at 550°C (heating rate: 1 K min^-1^) followed [58].

The amino modification of the silica surface was performed *via* post-grafting by dispersing 500 mg of NPSNPs in 20 mL toluene. To this dispersion 75 μL 1,8-diaza-bicyclo[5.4.0]undec-7-ene (DBU) and 95 μL 3-aminopropyltrimethoxysilane (APTMS) were added. Afterwards, the solution was stirred for 2 h at 80°C. The modified nanoparticles were collected by centrifugation, washed three times with ethanol and dried at 60°C [52].

### Immobilization and release of BDNF

BDNF immobilization took place in sterile solutions with a concentration of 1 μg mL^-1^ in phosphate-buffered saline (PBS, Sigma Aldrich, Munich, Germany), which contained 0.1% bovine serum albumin (BSA, Sigma Aldrich, Munich, Germany). BSA acted as stabilizer of BDNF and as filler protein to prevent protein adhesion to reaction tubes [59]. Recombinant BDNF was purchased from Life Technologies (Darmstadt, Germany). It was produced in *Escherichia coli* and had a purity higher than 98%.

5 mg of the nanoparticles, unmodified or amino-modified, were sterilized by shining UV light on the sample. Afterwards, the nanoparticles were incubated in 1 mL of the sterile protein solution for 24 h at 4°C in a Thermomixer (Biozym Scientific, Hessisch Oldendorf, Germany) under constant shaking of 1000 rpm. After the incubation, the samples were centrifuged and washed once with PBS (0.1% BSA). All incubation and washing solutions were kept frozen at -20°C to prevent any leakage of protein during storage for later analysis by ELISA. For control experiments 5 mg nanoparticles were treated under similar conditions, but without BDNF additive.

After incubation and washing, the release was started by giving 1 mL PBS (0.1% BSA) to the samples. PBS was chosen as release medium because it is an often used standard release medium to simulate physiological conditions and has a pH value of 7.4 similar to the cochlear fluid [60]. The samples were kept at 37°C. At various time intervals, the samples were centrifuged, the supernatants were removed and 1 mL fresh PBS (0.1% BSA) was added to continue the release. Similar to the incubation and washing solutions, all supernatants were frozen to be later analyzed by ELISA.

### Characterization methods

For the characterization of NPSNPs transmission electron microscopy (TEM) was performed. TEM images were taken on a FEI Tecnai G2 F20 TMP instrument (Hillsboro, USA) operated with 200 kV. For the preparation ethanolic or aqueous dispersions were dropped on Cu grids with a 400 mesh and dried overnight.

Dynamic light scattering (DLS) measurements were performed on a Zetasizer Nano ZSP from Malvern Instruments (Worcestershire, UK). The nanoparticles were redispersed in water with a concentration of 0.5 mg mL^-1^ by ultrasonification. Afterwards, the suspensions were transferred to a polystyrene cuvette.

Zeta potential titration curves were measured with a Zetasizer Nano ZSP and a MPT2 Autotitrator from Malvern Instruments (Worcestershire, UK). For each measurement disposable folded capillary cells (DTS1070) were used. Each sample was dispersed in water with a concentration of 0.5 mg mL^-1^. The curves were recorded on proceeding from basic to acidic pH by addition of HCl (0.2 M). At each pH value, the zeta potential was measured three times. For data analysis, Malvern Zetasizer Software Version 7.11 was used.

Nitrogen sorption measurements were performed at 77°K on a Quantachrome Autosorb-3 instrument (Boynton Beach, USA). The unmodified nanoparticles were outgassed in vacuum at 100°C and the amino-modified nanoparticles at 80°C for 24 h prior to the sorption measurements. To evaluate the data, the ASiQWin 2.0 software was used. Surface areas were estimated by applying the Brunauer-Emmett-Teller (BET) equation. The pore size distribution was calculated using non-linear density-functional theory (NLDFT) and fitting of the Quantachrome Kernel “N2 at 77 K on silica for cylinder pores, NLDFT equilibrium model” to the experimental data. Values for total pore volumes were estimated by the single point method at *p*/*p*_0_ = 0.92 to exclude interparticular volume.

### ELISA

For the quantification of the amount of immobilized and released BDNF an enzyme-linked immunosorbent assay (ELISA) kit against human BDNF (Boster Biological Technology Co., Ltd., Pleasanton, USA) was applied. The BDNF-ELISA kit was used in accordance to the manufacturer´s recommendations. In brief, a monoclonal antibody for BDNF had been precoated onto the 96-well plate. Standards and samples were diluted in sample dilution buffer and were added to the wells. After an incubation period of 90 min, the plate content was discarded and a working solution of a biotinylated anti-human BDNF antibody was added for 60 min. This was followed by three washing steps with PBS and by the incubation with a working solution containing an avidin-biotin-peroxidase complex. Then, the unbound conjugates were washed off with PBS. Finally, the detection was performed with 3,3´,5,5´-tetramethylbenzidine and stopped with 2 M sulphuric acid. All incubation steps were performed at 37°C. Absorbance was read at 450 nm on an EON spectrophotometer (Biotek, Winooski, USA).

### Cell culture investigations

#### NIH3T3 fibroblasts

The cell culture investigations were performed according to the investigations of Williams et al. [52]. The murine fibroblast cell line NIH3T3 (ATCC-Number: CRL-165) was used for the initial cytocompatibility tests. The nanoparticles were sterilized under UV light. For cultivation, nanoparticle stock solutions with a concentration of 1000 μg mL^-1^ were produced with sterile water. From these stock solutions the other tested concentrations of 10 μg mL^-1^, 100 μg mL^-1^, 250 μg mL^-1^ and 500 μg mL^-1^ were prepared by dilution with sterile water.

The NIH3T3 fibroblasts were cultivated in high glucose Dulbecco´s Modified Eagle´s Medium (DMEM, Biochrom, Berlin, Germany) with supplements like 10% fetal calf serum (FCS), 1% penicillin and streptomycin (Biochrom, Berlin, Germany). Cells were seeded with a density of 1 x 10^4^ cells per well in a 96-well plate (TPP, Trasadingen, Switzerland). The cultivation was performed for three days at 37°C in a humidified atmosphere (5% CO_2_) for expansion. After removing the medium, 50 μL fresh medium and 50 μL aqueous nanoparticle dispersion were given to the wells. So, the final concentrations which were tested are 5 μg mL^-1^, 50 μg mL^-1^, 125 μg mL^-1^, 250 μg mL^-1^ and 500 μg mL^-1^. Incubation took place for four days and every day the morphology and proliferation of the fibroblasts were checked with a transmission light microscope (CKX41, Olympus, Hamburg, Germany) with a CCD-camera (Colorview III, SIS, Olympus).

#### Neutral red uptake assay

After the incubation, the cell viability was determined by the neutral red uptake (NRU) assay. Neutral red (3-amino-7-dimethylamino-2-methylphenazine hydrochloride, Merck, Darmstadt, Germany) stock solution was prepared by dissolving 40 mg neutral red dye in 10 mL purified water (4 mg mL^-1^). The stock solution was diluted 1:50 in pre-heated (37°C) DMEM. The medium was removed and 100 μL of the neutral red medium were added per well. After an incubation of 3 h at 37°C and 5% CO_2_, the neutral red medium was discarded. Afterwards, the cells were washed and fixed by adding solution I (1% calcium chloride, 0.5% formaldehyde in purified water). After 5 min incubation time solution I was removed. Now, 100 μL of a neutral red destaining solution (1% acetic acid, 50% ethanol (95%) in purified water) were added. The plate was shaken and incubated for 10 min at 4°C. The absorption of the neutral red extract was measured at 570 nm using a microplate reader (Multiskan Ascent, Thermo Scientific Inc., Waltham, USA).

#### Ethics statement for isolation of SGCs from neonatal rats

The experiments and analysis of this study were conducted from October 2015 to September 2017. All experiments were carried out in accordance with the institutional guidelines for animal welfare of the Hannover Medical School following the standards described by the German ´Law on Protecting Animals´ (Tierschutzgesetz) and with the European Directive 2010/63/EU for protection of animals used for experimental purposes. For our *in vitro* experiments an euthanasia was used, which is registered (no.:2013/44) with the local authorities (Zentrales Tierlaboratorium, Laboratory Animal Science, Hannover Medical School, including an institutional animal care and use committee) and reported on a regular basis as demanded by law. For exclusive sacrifice of animals for tissue analysis in research, no further approval is needed if no other treatment is applied beforehand (§4). The rats were bred and born for research study purposes. A breeding stock was supplied by Charles River (Charles River, Wilmington, USA) and housed with their litters in the facilities of the licensed Institution of Laboratory Animal Science of the Hannover Medical School. To minimize the stress level for the neonatal rats, they were rapidly decapitated prior to any experimentation by a licensed person.

#### Spiral ganglion cells

In the cell culture investigations with spiral ganglion cells (SGCs), the neuroprotective action of amino-modified nanoparticles with immobilized BDNF and of released BDNF amounts was investigated. SGCs, obtained by dissociation of the spiral ganglion, provide mixed cultures containing neurons, fibroblasts and glial cells. The primary SGCs were isolated from neonatal Sprague-Dawley rats (postnatal day 3–5). Rats were sacrificed by rapid decapitation. The dissection of the cochleae and the enzymatic and mechanical dissociation of the spiral ganglia were performed according to a previously described protocol [61,62]. Afterwards, the number of viable cells was determined using a Neubauer chamber (Brand, Wertheim, Germany) and the trypan blue staining (Sigma Aldrich, Munich, Germany). The dissociated cells were seeded at a density of 1 x 10^4^ cells per well in a 96-well plate (TPP). Prior to cell seeding, the used plates were coated with poly-D/L-ornithine (0.1 mg mL^-1^, Sigma Aldrich, Munich, Germany) and laminin (0.01 mg mL^-1^, Life Technologies, Carlsbad, USA). 50 μL of the tested samples (nanoparticle dispersion or released supernatants) and 50 μL fresh medium were given to the wells. The incubation was performed for 48 h in serum-free medium (Panserin 401, PAN Biotech, Aidenbach, Germany), which was supplemented with HEPES (25 mM, Life Technologies, Carlsbad, USA), glucose (6 mg mL^-1^, Braun AG, Melsungen, Germany), penicillin (30 U mL^-1^, Grünenthal GmbH, Aachen, Germany), N2-supplement (3 μg mL^-1^, Life Technologies, Carlsbad, USA) and insulin (5 μg mL^-1^, Sigma Aldrich, Munich, Germany). After the incubation, the SGCs were fixed with a 1:1 methanol (Carl Roth, Karlsruhe, Germany) and acetone (J.T. Baker, Arnhem, Netherlands) solution and washed with PBS (Gibco® by Life Technologies, Carlsbad, USA). In all experiments, we included a negative control (SGCs cultivated in serum-free medium), a positive control (SGCs in medium with 50 ng mL^-1^ BDNF additive), an additional control of a solution of SGC medium/PBS (0.1% BSA) (1:1), which were also incubated for 48 h, and a control of the seeding density after 4 h cultivation.

#### Survival rate and neurite length of SGNs

The cultures from dissociated SGCs consist of neurons, fibroblasts and glial cells. For the identification of SGNs from the superior number of other cells, a monoclonal mouse 200 kDa-neurofilament antibody (clone RT97, Leica Biosystems, Wetzlar, Germany) was used as a neuron-specific marker. Briefly, fixed cells were incubated with the primary neurofilament antibody. After washing with PBS, the cells were incubated with a secondary biotinylated anti-mouse antibody (Vector Laboratories Inc., Burlingame, USA). Before the staining was visualized with diaminobenzidine (Peroxidase Substrate Kit DAB, Vector Laboratories Inc., Burlingame, USA), the cells were incubated with an ABC complex solution (Vectastain Elite ABC-Kit, Vector Laboratories Inc., Burlingame, USA) according to the manufacturer´s protocol [62]. Finally, the samples were imaged with an inverted microscope (CKX41, Olympus) and the number of survived SGNs was determined. Surviving neurons were defined as neurofilament-positive cells with a neurite length of at least three cell soma diameters [21]. The survival rate was calculated by relating the number of survived neurons per well to the seeding density of the control sample in the same plate after 4 h cultivation. To examine the neuroregenerative effect of the nanoparticle dispersion and the released supernatants of NPSNPs, the five longest neurons in each field of view (one in the centre and four around the perimeter of the well) were imaged using a transmission light microscope (Olympus CKX41) with a CCD-camera (Colorview III, SIS, Olympus). Finally, the length of the neurons was measured by using the imaging software CellP (SIS). Here, the conditions were blinded for the analyst.

#### Statistical analysis

Statistical analysis was performed with Prism 5 (GraphPad, La Jolla, USA). The results were validated by using one-way analysis of variance (ANOVA) followed by Bonferroni´s multiple comparison test. *P* values of less than 0.05 were considered to be statistically significant. All quantitative data represent the means of at least two independent approaches (*N*), including at least triplicates of each sample (*n*). Error bars in the figures indicate the standard error of the mean. Levels of significance are indicated as follows: n.s. = not significant, *p<0.05; **p<0.01; ***p<0.001.

## Results and discussion

### Characterization of the nanoporous silica nanoparticles

**Fig 1:**
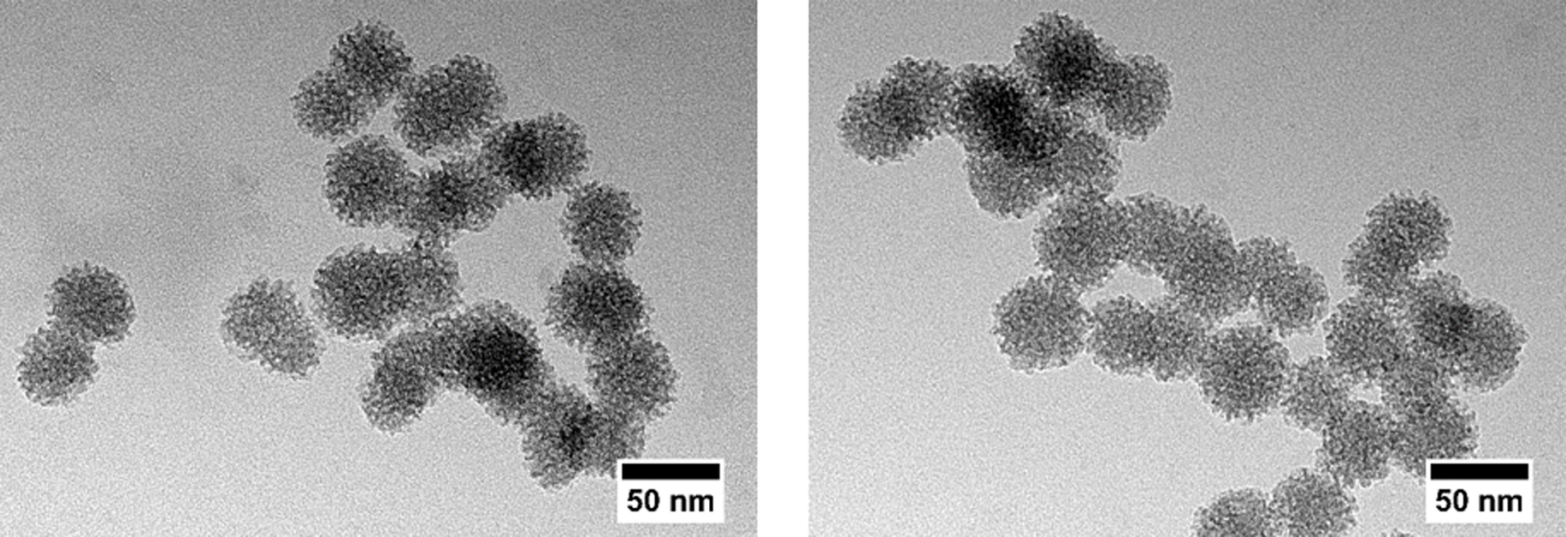
TEM images of the unmodified (left) and amino-modified (right) NPSNPs.

To investigate the size and morphology of the synthesized nanoparticles, TEM images were taken. TEM images of the unmodified (Fig 1, left) and the amino-modified nanoparticles (Fig 1, right) reveal spherical nanoparticles with a diameter of ca. 45 nm and a narrow size distribution. DLS measurements of both types of nanoparticles (shown in S1 Fig) support these results and show that the nanoparticles are only slightly agglomerated. The TEM images also show the high degree of porosity of both types of nanoparticles. The nanoporosity was further quantified by nitrogen sorption measurements. The BET surface area calculated from the isotherms (shown in S2 Fig) amounts to 1160 m^2^ g^-1^ for the unmodified nanoparticles, a value which decreases to 440 m^2^ g^-1^ for the amino-modified nanoparticles; the pore volume decreases as well, from 1.0 cm^3^ g^-1^ to 0.5 cm^3^ g^-1^. Nevertheless, the values show that a high porosity is still present and the average pore size for both types of nanoparticles is similar with 3.1 nm. The decrease of the surface area and the pore volume of the amino-modified nanoparticles (while the pore diameter remains unaffected) can be explained by a partial pore blocking which might result either from a slight dissolution/reprecipitation of the nanoparticles during the modification treatment or from the deposition of organosilane moieties on the particle surface. Such moieties can form in solution by self-condensation of the silanization reagent.

**Fig 2:**
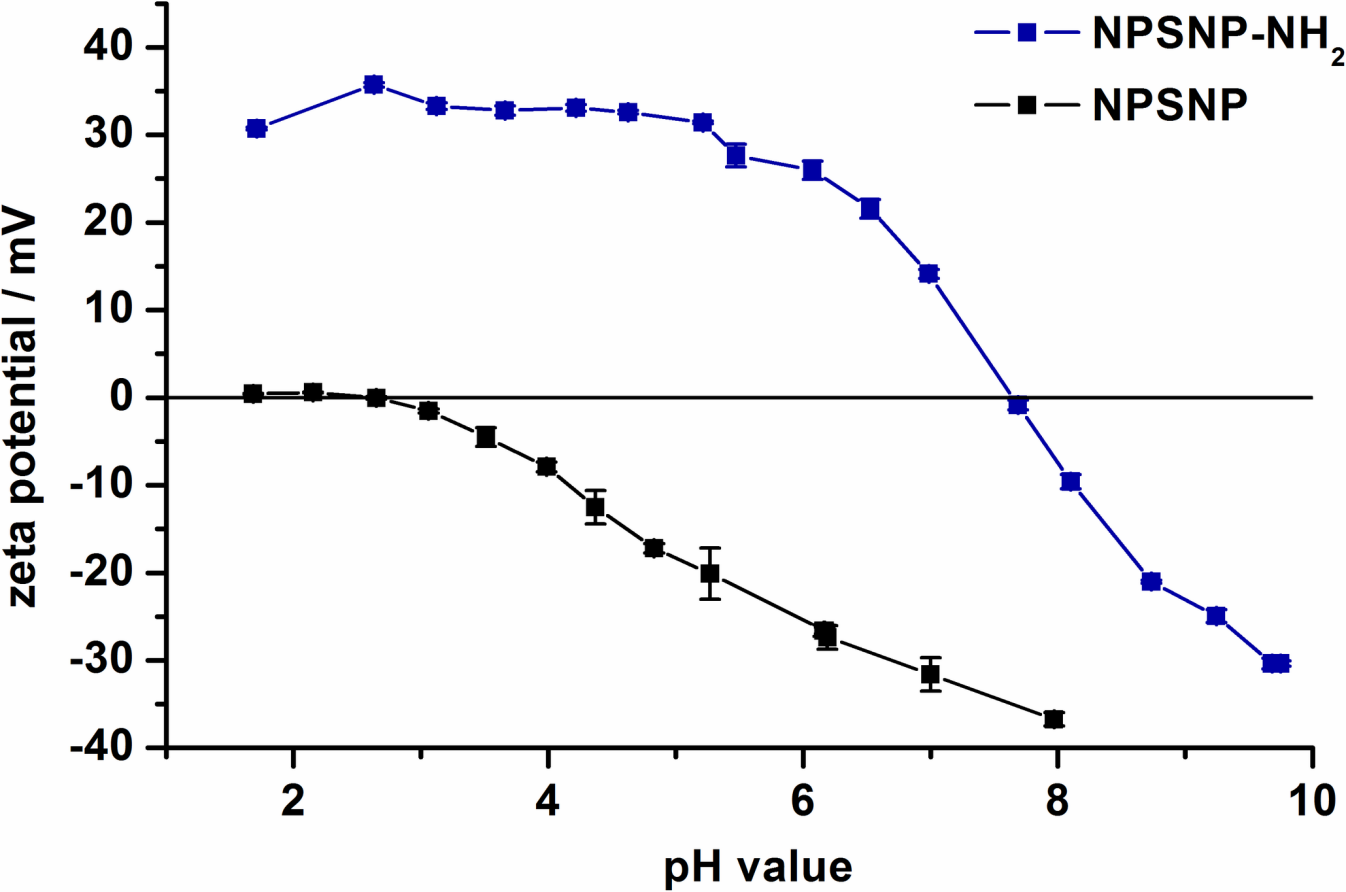
Zeta potential titration curves for the unmodified NPSNPs in comparison to the amino-modified NPSNPs.

The success of the amino modification was confirmed by pH-dependent zeta potential measurements (Fig 2). The amino modification of the nanoparticles leads to a different titration curve of the pH-dependent zeta potential. The unmodified nanoparticles show a negative zeta potential up to a pH value of 2.6. At lower pH values the nanoparticles show a nearly neutral zeta potential. This behavior is typical for silica nanoparticles due to the weakly acidic silanol groups on the silica surface [63]. The amino-modified nanoparticles reach the isoelectric point at a pH value of 7.7 and have a strongly positive zeta potential at lower pH values. The change in the zeta potential is caused by the protonation of the amino groups.

### Quantification of BDNF loading and release

To quantify the amounts of immobilized BDNF, ELISAs were carried out. The immobilized BDNF amount was calculated indirectly by determining the remaining concentrations of BDNF in the incubation solution and in the washing solution (Table 1). The concentrations of BDNF in these solutions were very small (in the low ng mL^-1^ range vs. 1 μg mL^-1^ in the incubation solution). Thus, according to the ELISA results, both types of nanoparticles appear to have immobilized nearly the total amount of BDNF which was used in the incubation, as shown by the low BDNF concentrations in the remaining incubation and washing solutions. However, caution is advised in interpreting these results due to the possibility that some protein molecules may have undergone conformational changes in solution or upon ad- and desorption to the nanoparticle surface; these are no longer immunologically active and do not contribute to the ELISA signal. So, the indirectly determined immobilization amounts may be too high. In spite of the high apparent loading efficiency, we think that most of the protein was adsorbed on the outer surface of our NPSNPs, because the nanopores with a size of ca. 3 nm are probably too small for the encapsulation of protein molecules.

**Table 1:** Quantification of the amount of immobilized BDNF on unmodified and amino-modified NPSNPs which were incubated in a solution containing 1 μg mL^-1^ BDNF.

Nanoparticle type	Remaining incubation solution (ng mL^-1^)	Washing solution (ng mL^-1^)	Apparent loading efficiency (%)
NPSNP	4.1±3.4	4.0±3.7	99.1
NPSNP-NH_2_	2.1±0.9	1.8±0.1	99.6

**Fig 3:**
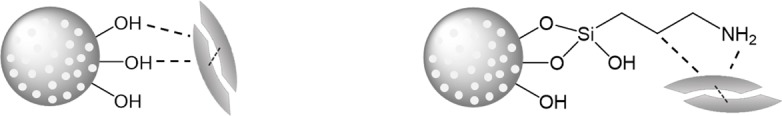
Scheme of the possible interactions between BDNF with (left) unmodified NPSNPs and (right) amino-modified NPSNPs. With unmodified NPSNPs, BDNF can interact via hydrogen bonds and electrostatic interactions, and with amino-modified NPSNPs via hydrogen bonding, electrostatic interactions and via hydrophobic effects. This schematic view does not show the nanoparticles, atoms and the protein with their real sizes.

The immobilization of the protein on both types of nanoparticles can be attributed to different protein-surface interactions (Fig 3). The unmodified nanoparticles can interact with BDNF electrostatically and via hydrogen bonds. In PBS at a pH value of 7.4, the nanoparticles show a negative zeta potential and BDNF, as a basic protein with an isoelectric point of approximately 10, has a net positive charge [18,42,64,65]. In addition, the ample silanol groups on the surface can interact with corresponding groups of the protein via hydrogen bonds. In contrast, the amino-modified nanoparticles are positively charged at a pH value of 7.4. Probably, interactions occur with negatively charged domains on the surface of the protein molecules. Hydrophobic interactions between the propyl chains of the aminopropyl moieties and nonpolar regions of the surface of the protein molecules presumably add up [66,67,68].

**Fig 4:**
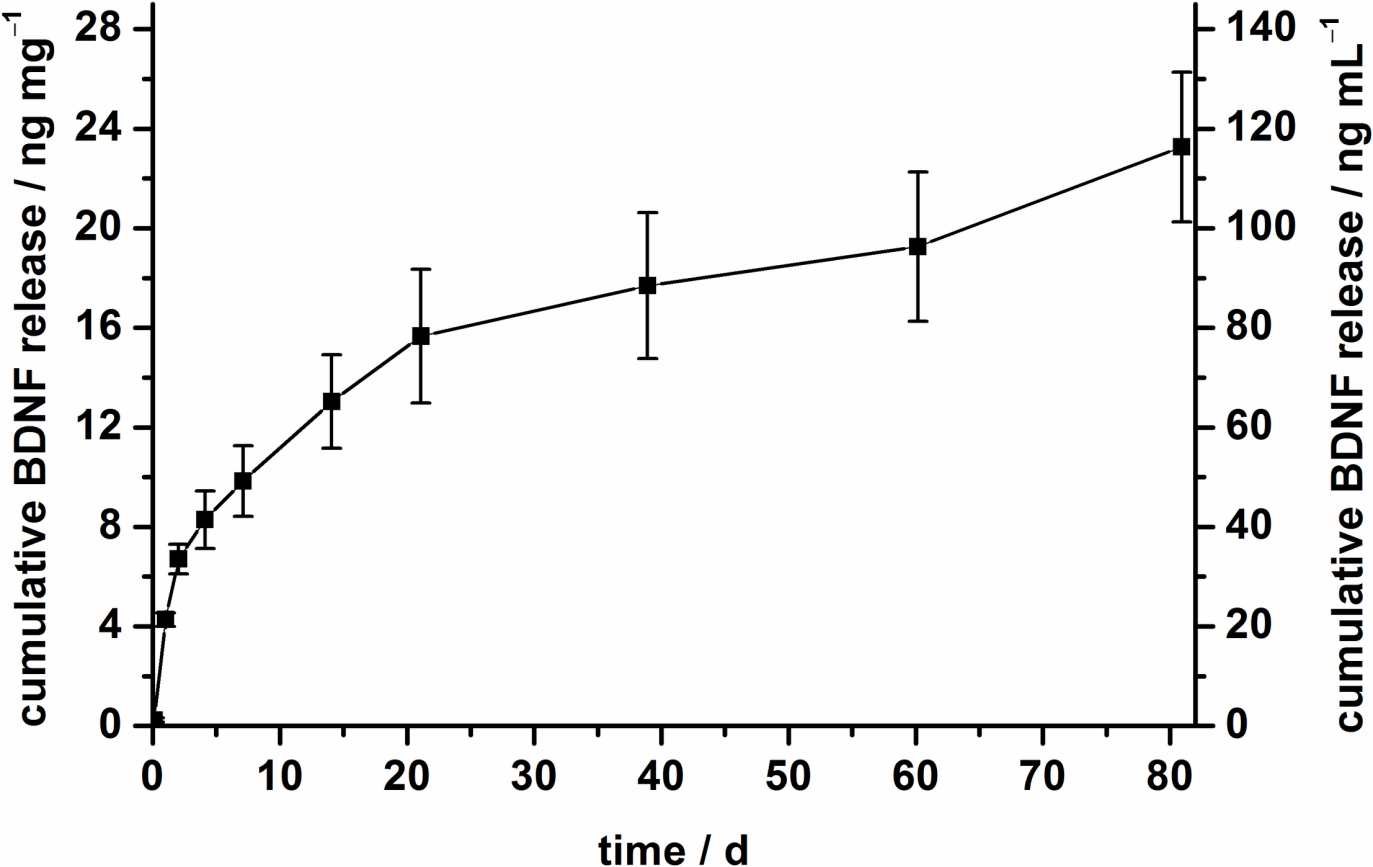
BDNF release profile of amino-modified NPSNPs in PBS (0.1% BSA) over 80 days at 37°C. The left axis represents the cumulative BDNF release with regard to 1 mg of nanoparticles and the right axis shows the cumulative release of BDNF referred to 1 mL release medium.

The release profiles of BDNF were investigated by incubating 5 mg nanoparticles in PBS with 0.1% BSA at 37°C. The amounts of released BDNF were determined by ELISA. The cumulative release of BDNF from amino-modified nanoparticles over a period of 80 days is depicted in Fig 4. A sustained release was observed and after 80 days, 23 ng mg^-1^ were released from the modified nanoparticles. In total, approximately 11% of the apparently immobilized amount of BDNF (based on the indirect determination) were released, but the release curve indicates that the release is not finished after 80 days. The driving force for the BDNF release is the concentration gradient of BDNF between the nanoparticle surface and the surrounding medium. Moreover, the well-known dissolution of nanoporous silica nanoparticles under physiological conditions like PBS can lead to an additional release of BDNF [69,70]. A partial dissolution of the NPSNPs can be observed after the release period of 80 days, because a qualitative decrease in the density of the dispersions of the NPSNPs is visible. This observation can be confirmed by the TEM investigations (S3 Fig) of the nanoparticles after simulated release periods of 7 d and 14 d. The nanoparticles are visibly smaller and the visibility of the pore system seems to be less pronounced, in line with the assumption that dissolution and possibly reprecipitation processes occur in aqueous solution at 37°C.

**Fig 5:**
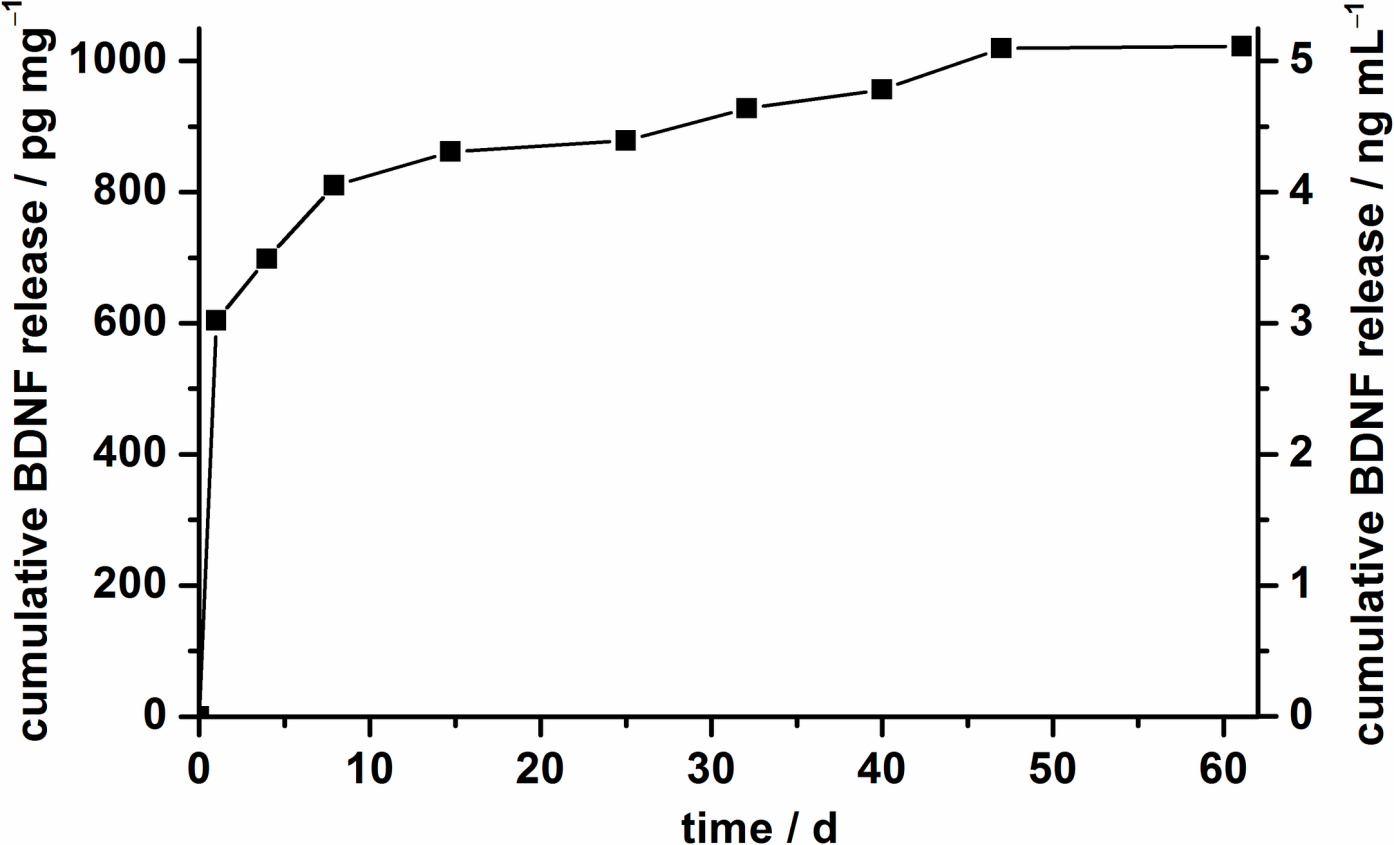
BDNF release profile of unmodified NPSNPs in PBS (0.1% BSA) over 60 days at 37°C. The left axis represents the cumulative BDNF release with regard to 1 mg of nanoparticles and the right axis shows the cumulative release of BDNF referred to 1 mL release medium.

In comparison, the BDNF release from unmodified nanoparticles was already finished after 60 days (Fig 5). Also the release behavior is different from that of the amino-modified nanoparticles. After a burst release on the first day, only small amounts of BDNF were delivered later. In the total release period, only 1 ng mg^-1^ BDNF was released. The different release behaviors should be attributable to the different interactions of the nanoparticles with the protein, but the small released amounts from the unmodified nanoparticles cannot be explained yet, especially due to the fact that both nanoparticle types show apparently high loading efficiencies. Moreover, previous studies exhibited a successful release of BDNF from silica supraparticles build up from unmodified nanoporous silica nanoparticles [43]. Nevertheless, according to our results on BDNF release, we propose to employ amino-modified NPSNPs for the immobilization of BDNF, as they offer a sustained long-term delivery of BDNF; therefore, we have chosen this type of particles for cell-culture studies of SGCs.

### Cell culture investigations

The cytocompatibility and the biocompatibility of NPSNPs have been thoroughly investigated in previous studies [49–53,71,72]. Nonetheless, we present tests on the general cytocompatibility of our prepared nanoparticles for NIH3T3 fibroblasts as a standard cell line. However, the main focus lays on the cell culture investigations with spiral ganglion cells (SGCs), including the primary auditory neurons of the inner ear, the spiral ganglion neurons (SGNs). Firstly, it is important to investigate the interaction of SGNs with NPSNPs to make statements about the cytocompatibility of the nanoparticles for these cells, especially with regard to the possible application of BDNF-loaded NPSNPs in the inner ear. Finally, we also plan to fix the particles on the cochlear implant; this is a scenario, where the loaded NPSNPs are not in direct contact with the SGCs, so that only BDNF actually released from the nanoparticles can exert a biological action. Thus, we also investigated the neuroprotective effect of solutions into which BDNF had been released in an independent prior experiment. To the best of our knowledge, the neuroprotective effect of such previously released BDNF on SGNs has not been investigated so far in *in vitro* experiments.

#### Fibroblast cell culture studies

**Fig 6:**
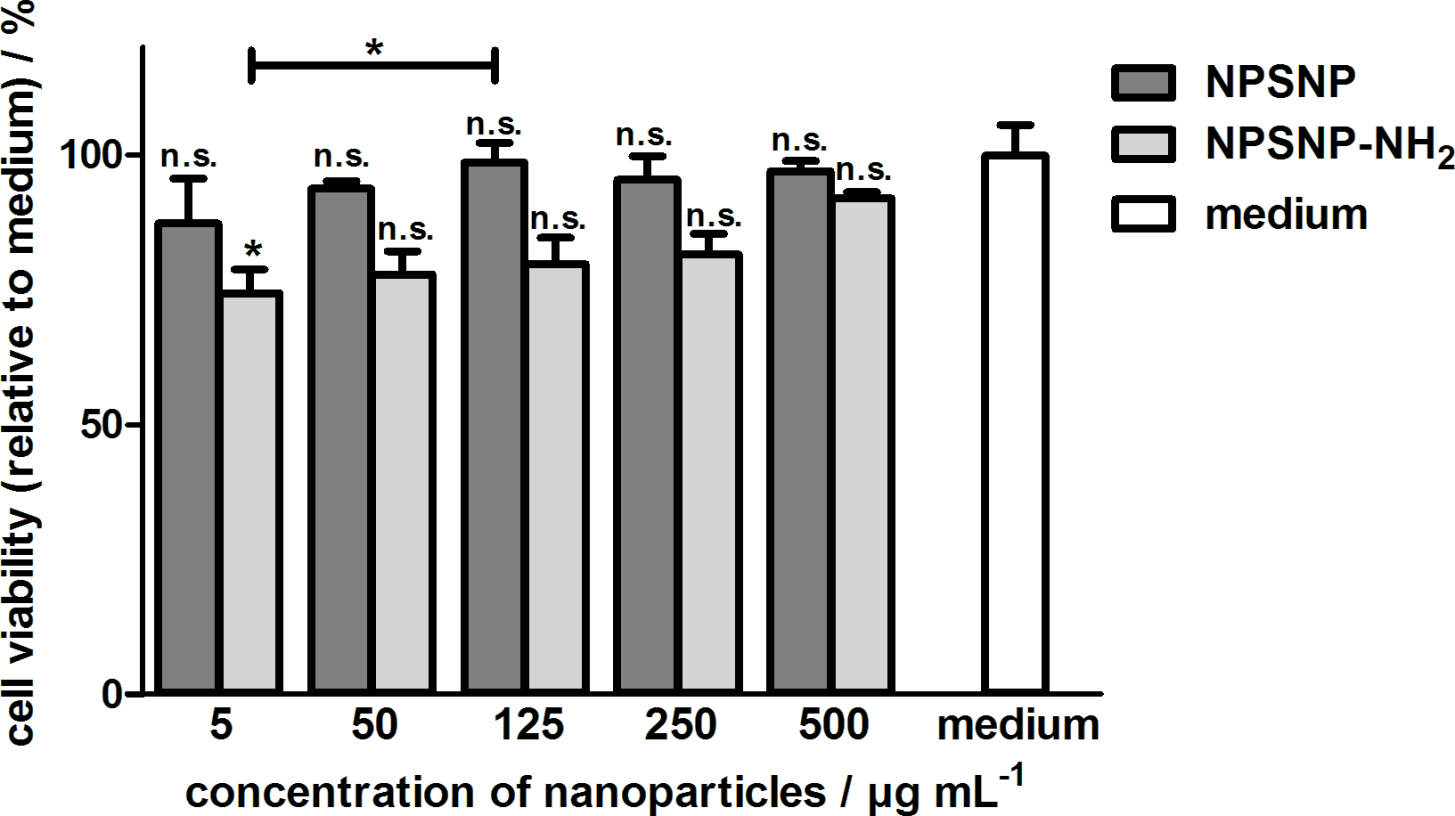
Relative cell viabilities of NIH3T3 fibroblasts in the presence of unmodified and amino-modified nanoparticles in different concentrations determined by the NRU assay after an incubation for four days. Values are given as mean ± standard error of the mean (*n* = 3).

Fibroblasts were cultivated in the presence of both types of nanoparticles in different concentrations to obtain general information regarding the cytocompatibility of the prepared nanoparticles. To determine the cell viability, the NRU assay was used. Results are shown in Fig 6. These indicate that unmodified and amino-modified nanoparticles are cytocompatible for fibroblasts due to the fact that the relative cell viability is above 70% in all cases, a defined criterion for cytocompatibility according to DIN EN ISO 10993–5 [73]. Noteworthy, good cytocompatibility is also obtained for very high concentrations up to 500 μg mL^-1^. Only a slightly better cell viability for the unmodified nanoparticles was obtained. These results confirm our previous study that had already shown a good cytocompatibility of unmodified and amino-modified nanoparticles for NIH3T3 fibroblasts and for HepG2 cells (human hepatocellular carcinoma cell line) [52,53].

#### Spiral ganglion cell culture studies with BDNF-loaded NPSNPs

An important goal is to determine the suitability of NPSNPs as new carrier system for delivering neurotrophins safe and in an effective concentration over the long term. Thus, the effect of the nanoparticles on the survival of the SGNs, the primary auditory neurons in the cochlea, is of importance. For this purpose, the survival rate of the SGNs in presence of amino-modified nanoparticles with and without immobilized BDNF was tested (the unmodified nanoparticles were not considered further due to the less promising results in release studies). Two concentrations of the dispersions of amino-modified nanoparticles (250 μg mL^-1^ and 500 μg mL^-1^), which have shown a good cytocompatibility with fibroblasts, were chosen. The nanoparticle dispersion in PBS (0.1% BSA) was mixed 1:1 with SGC medium to get final concentrations of 125 μg mL^-1^ and 250 μg mL^-1^. Finally, the SGCs were cultivated for two days in these mixtures.

**Fig 7:**
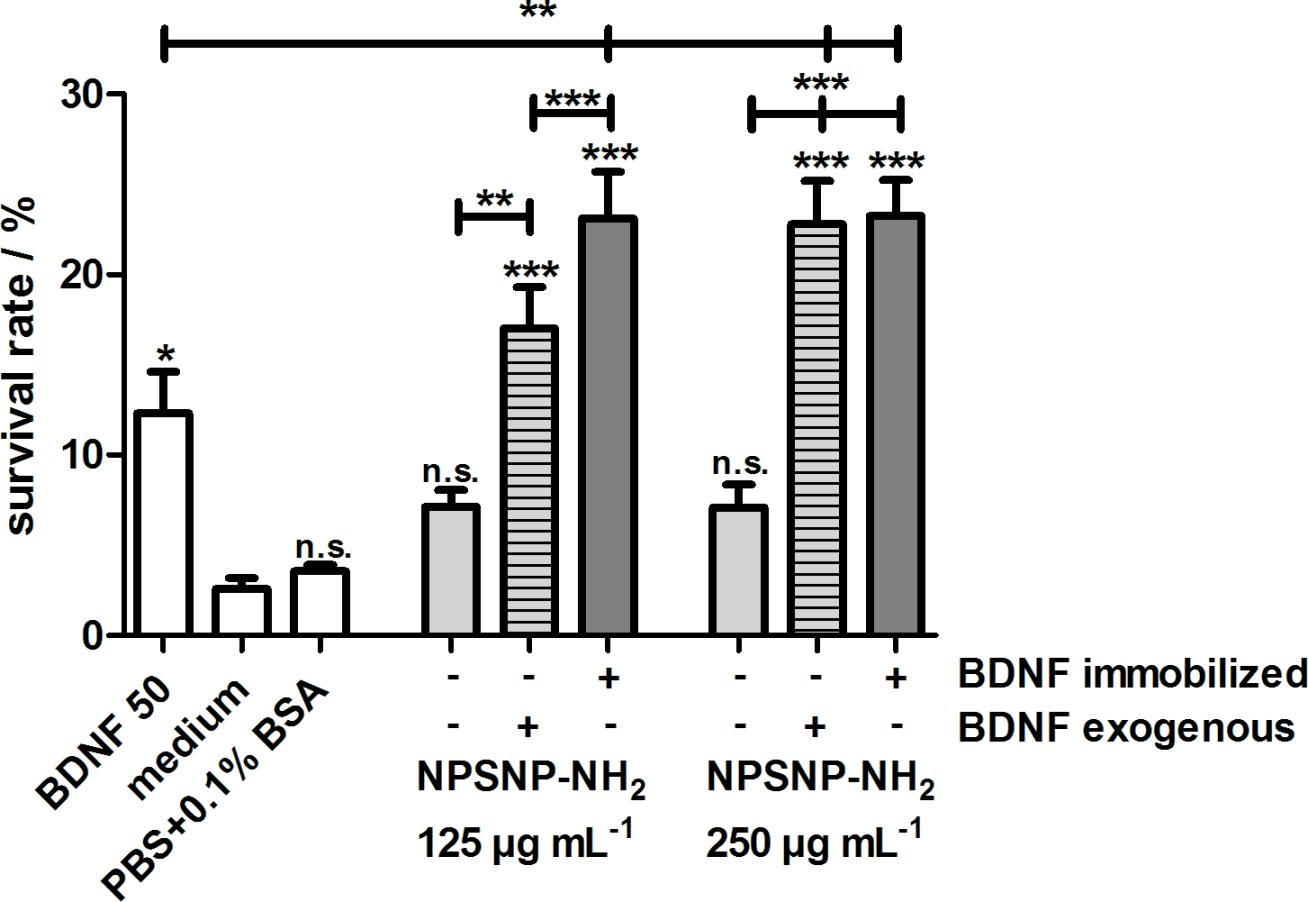
Comparison of the survival rates of spiral ganglion neurons after 48 h hof cultivation. Cells were cultivated in the presence of amino-modified nanoparticles (NPSNP-NH_2_; light grey), BDNF-loaded amino-modified nanoparticles (dark grey) and amino-modified nanoparticles with an exogenous addition of 50 ng mL^-1^ BDNF (light grey, striped). Values are given as mean ± standard error of the mean (*N* = 2, *n* = 3). Statistical assessment was performed using one-way ANOVA with Bonferroni´s multiple comparison test (n.s. = not significant, *p<0.05; **p<0.01; ***p<0.001). Asterisks over the bars indicate the significance of the survival rates of different conditions compared to the negative control (serum-free SGC medium). Asterisks between two bars indicate the significance between the respective conditions.

After the cultivation under different conditions, the number of survived neurons in relation to the seeding control, i.e., the survival rates, were determined using an inverted microscope. The values for the survival rates of SGNs are shown in Fig 7. The survival rates of SGNs for the BDNF-free nanoparticle dispersions of both concentrations were slightly higher than for the control of a 1:1 SGC medium/PBS (0.1% BSA) solution. According to that, the amino-modified nanoparticles themselves appear to have a slight neuroprotective effect on SGNs. This fact is supported by the significantly higher survival rate in the presence of 250 μg mL^-1^ BDNF-free nanoparticles with additional exogenous BDNF supply of 50 ng mL^-1^ as compared to the survival rate in the presence of 50 ng mL^-1^ BDNF only. To the best of our knowledge, such a neuroprotective effect of (amino-modified) silica nanoparticles has not been reported so far. This aspect generally favors the usage of such nanoparticles for applications in the inner ear and deserves further investigation. More relevant is the aspect that the survival rates of SGNs were significantly higher in the presence of BDNF-loaded nanoparticles, regardless of the concentration, when compared to the unloaded nanoparticles. More precisely, the survival rates for BDNF-loaded nanoparticle dispersions with concentrations of 125 μg mL^-1^ and 250 μg mL^-1^ were 23.1% ± 2.6% and 23.3% ± 2.0%, respectively, and were the highest in this experiment. In fact, they were statistically significantly higher than the value of the positive control, which is the SGC medium supplemented with 50 ng mL^-1^ recombinant BDNF (p<0.01).

In addition, we included in our experiments a comparison between cell cultures where we added either BDNF-loaded NPSNPs or BDNF-free NPSNPs and an additional exogenous BDNF supply of 50 ng mL^-1^. This concentration was chosen because the positive control also contained 50 ng mL^-1^ BDNF. Interestingly, BDNF that was immobilized on the NPSNPs is at least as effective (for the nanoparticle concentration of 250 μg mL^-1^) or even more effective (for the nanoparticle concentration of 125 μg mL^-1^) than the exogenously applied BDNF. This is surprising since the released concentration of BDNF from NPSNPs after two days of cultivation amounts to approximately 0.8 ng mL^-1^ or 1.6 ng mL^-1^, respectively, depending on the concentration of the nanoparticles. Consequently, and importantly, the BDNF immobilized on the NPSNPs has to a sufficient part retained its biological activity in order to have a positive effect on the survival of the SGNs.

**Fig 8:**
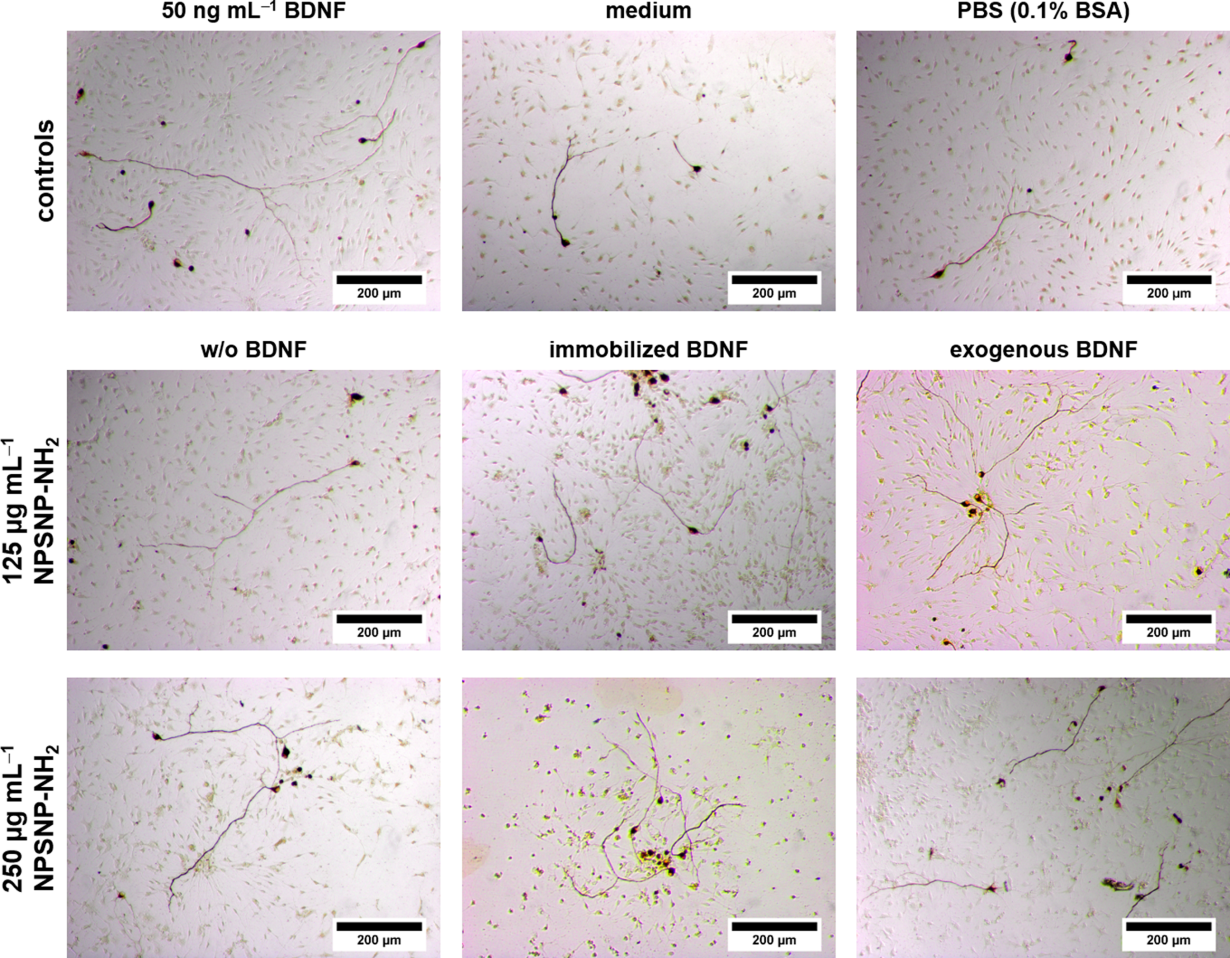
Representative microscopic images of spiral ganglion cell cultures cultivated for 48 h. The cells were cultivated in the presence of amino-modified nanoparticles (w/o BDNF), BDNF-loaded amino-modified nanoparticles (immobilized BDNF) and amino-modified nanoparticles with an exogenous addition of 50 ng mL^-1^ BDNF (exogenous BDNF) in two different concentrations. For comparison, SGCs were also cultivated in serum-free SGC medium (medium) and in serum-free medium supplemented with BDNF (50 ng mL^-1^ BDNF) as well as in a serum-free medium/PBS solution (1:1) (PBS (0.1% BSA)).

Representative microscopic images support the results obtained from the comparison of the SGN survival rates. After two days of cultivation, in all conditions improved neuronal survival was observed (Fig 8), but there are visible differences in the number of survived SGNs. As expected, all cell cultures with BDNF, whether immobilized or exogenously added, showed a higher number of neurons.

**Fig 9:**
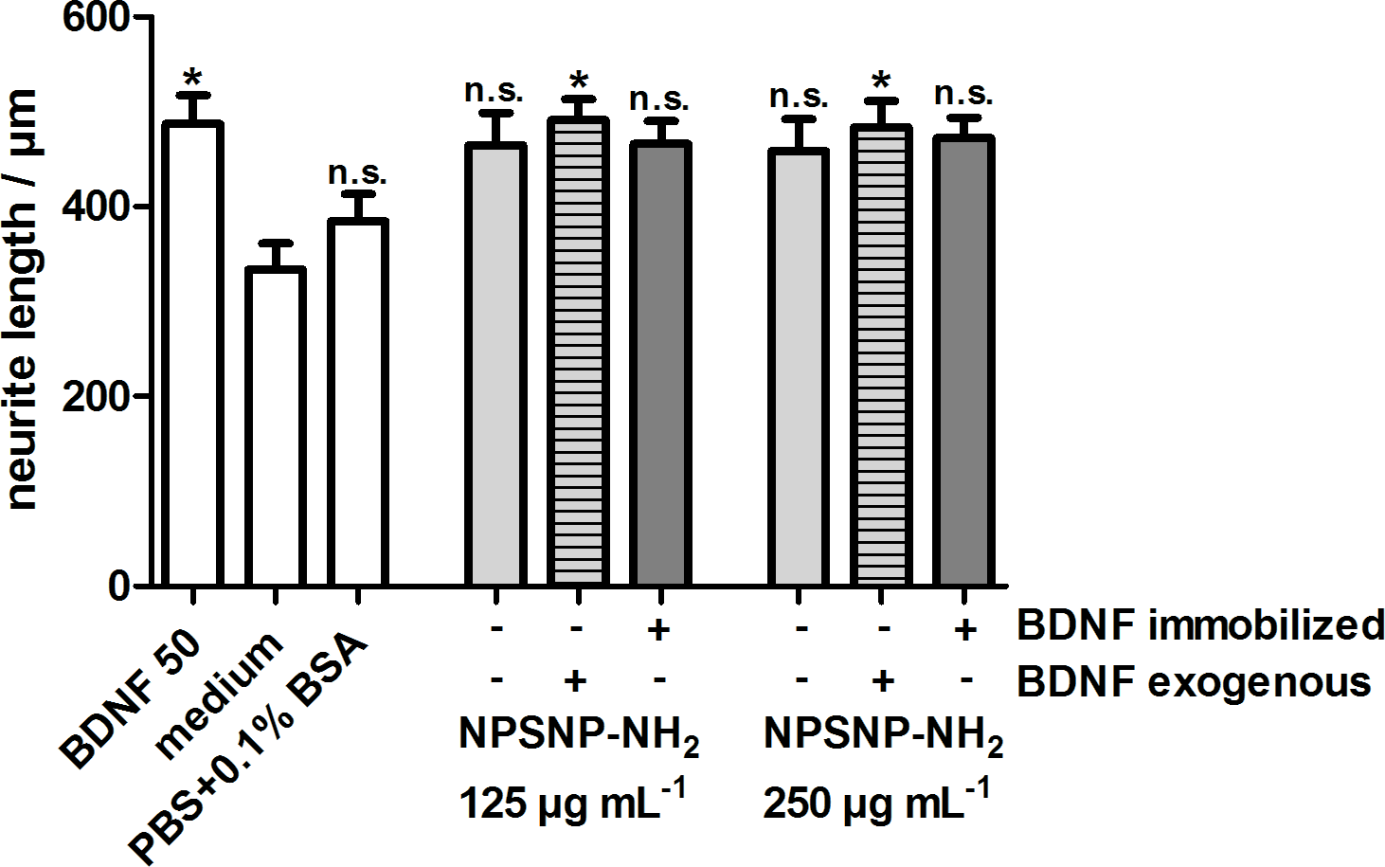
Neurite length of SGNs cultivated with BDNF-loaded (dark grey) and BDNF-free NPSNPs with (light grey, striped) or without (light grey) an additional exogenous BDNF supply. Values are given as mean ± standard error of the mean (*N* = 2, *n* = 3). Statistical assessment was performed using one-way ANOVA with Bonferroni´s multiple comparison test (n.s. = not significant, *p<0.05; **p<0.01; ***p<0.001). Asterisks over the bars indicate the significance of the survival rates of the different conditions compared to the medium control (serum-free SGC medium).

In order to quantify a possible neuroregenerative effect, we determined the neurite lengths of the SGNs which had survived. The results are depicted in Fig 9. The cultivation of SGNs with BDNF-loaded NPSNPs and BDNF-free NPSNPs with or without an additional exogenous BDNF supply led to an increased neurite length (ranging from 422 ± 36 μm to 491 ± 22 μm) in comparison to the negative control (334 ± 27 μm). Interestingly, BDNF-loaded and BDNF-free nanoparticles both apparently show a slight neuroregenerative effect, although this is significant only when BDNF was added exogenously. However, no differences regarding the neurite lengths are visible between the BDNF-loaded and BDNF-free nanoparticles.

The *in vitro* results for the interaction of BDNF-loaded silica nanoparticles with SGNs correlate quite well with previous studies. *In vivo* experiments have already shown that BDNF-loaded supraparticles based on nanoporous silica nanoparticles can improve SGN survival. The supraparticles were injected via a cochleostomy into the basal turn of deafened guinea pigs [10,43]. In contrast to our delivery system, BDNF was encapsulated in larger pores of the used silica nanoparticles [74] and between the nanoparticles making up the supraparticles. This is not necessarily an advantage compared to the immobilization of BDNF on the nanoparticle surface like Pakulska et al. have shown [65]. A further study indicated the overall safety of silica nanoparticles for the use in the inner ear, because no adverse effects on hearing or cochlear function were noted. In this case, nanoparticles were applied onto the round window membrane, as usually would be performed also in a clinical setting, penetrated into the cochlea and were even taken up into the spiral ganglion neurons [55].

#### Spiral ganglion cell culture studies with released BDNF

**Fig 10:**
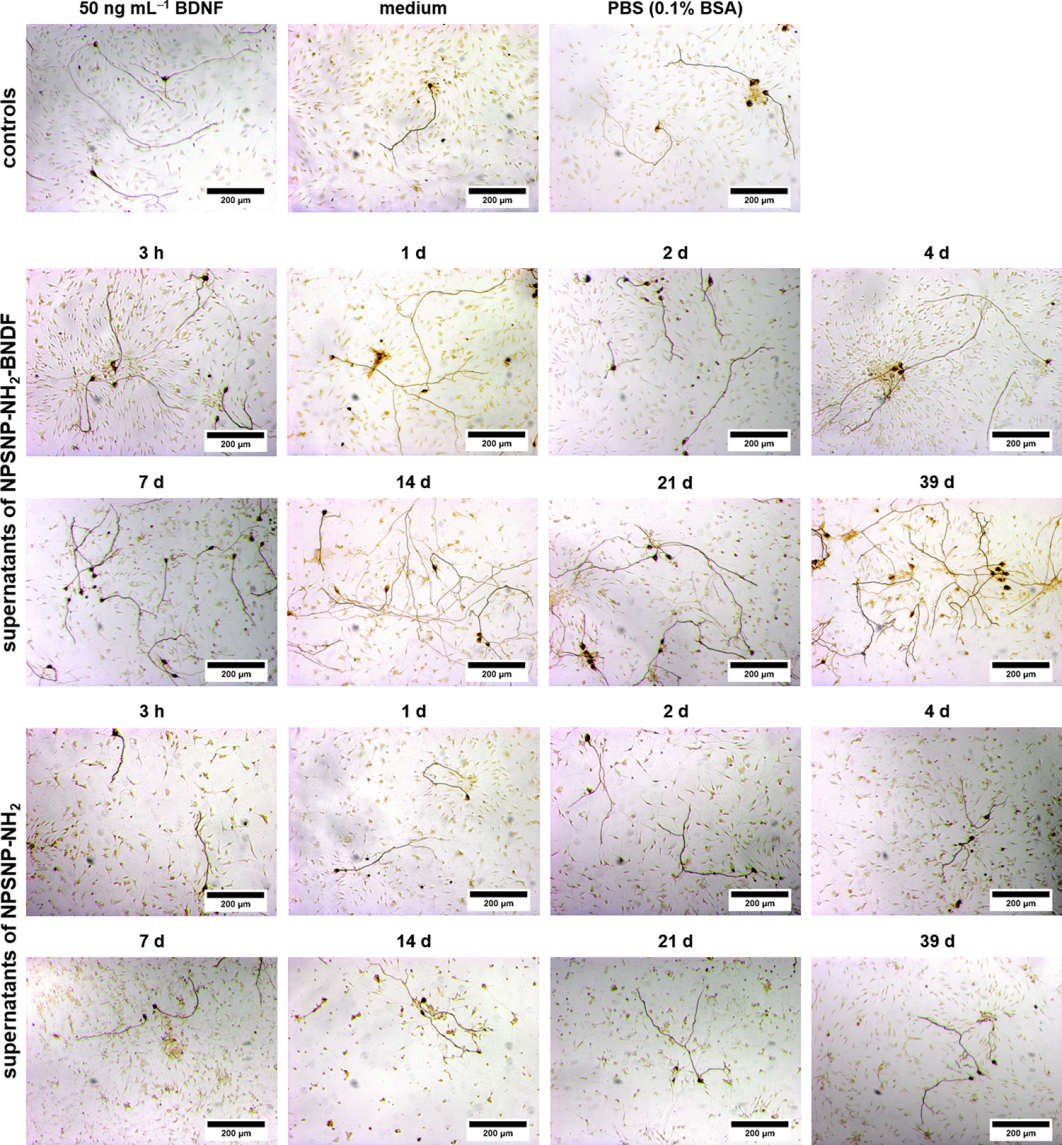
Microscopic images of spiral ganglion cells (SGCs) after two days of cultivation in different media composition. The cells were cultivated in presence of the supernatants from the release experiments of BDNF-loaded amino-modified NPSNPs (NPSNP-NH_2_-BDNF) or of BDNF-free amino-modified NPSNPs (NPSNP-NH_2_). For comparison, SGCs were also cultivated in serum-free SGC medium (medium) and in serum-free medium supplemented with BDNF (50 ng mL^-1^ BDNF) as well as in a 1:1 serum-free medium/PBS (0.1% BSA) solution.

In spite of the informational value of the experiments described above, the situation in the finally envisaged application *in vivo* is different. There, the target cells are not in direct contact to the delivering nanoparticles since the implant-associated delivery system is at some distance from the spiral ganglion cells (SGCs), and BDNF can only act on these cells after release from the drug delivery system. In order to closer match the clinical situation, additional *in vitro* experiments were carried out using supernatants rather than the NPSNPs. In this context, SGCs were cultured in supernatants obtained from release experiments with 5 mg mL^-1^ BDNF-loaded or unloaded nanoparticles. Thus, only BDNF that was released from NPSNPs can exert its action. The supernatants were frozen until they were added to the SGC culture. The cells were cultivated for two days in a 1:1 mixture of serum-free SGC medium and the collected supernatant. The microscopic images obtained are shown in Fig 10 and demonstrate great differences regarding the neuronal survival. In the cell cultures of the supernatants from NPSNP-NH_2_-BDNF, a high number of neurons was clearly observed. In comparison, the control supernatants from BDNF-free nanoparticles (NPSNP-NH_2_) show less neurites.

**Fig 11:**
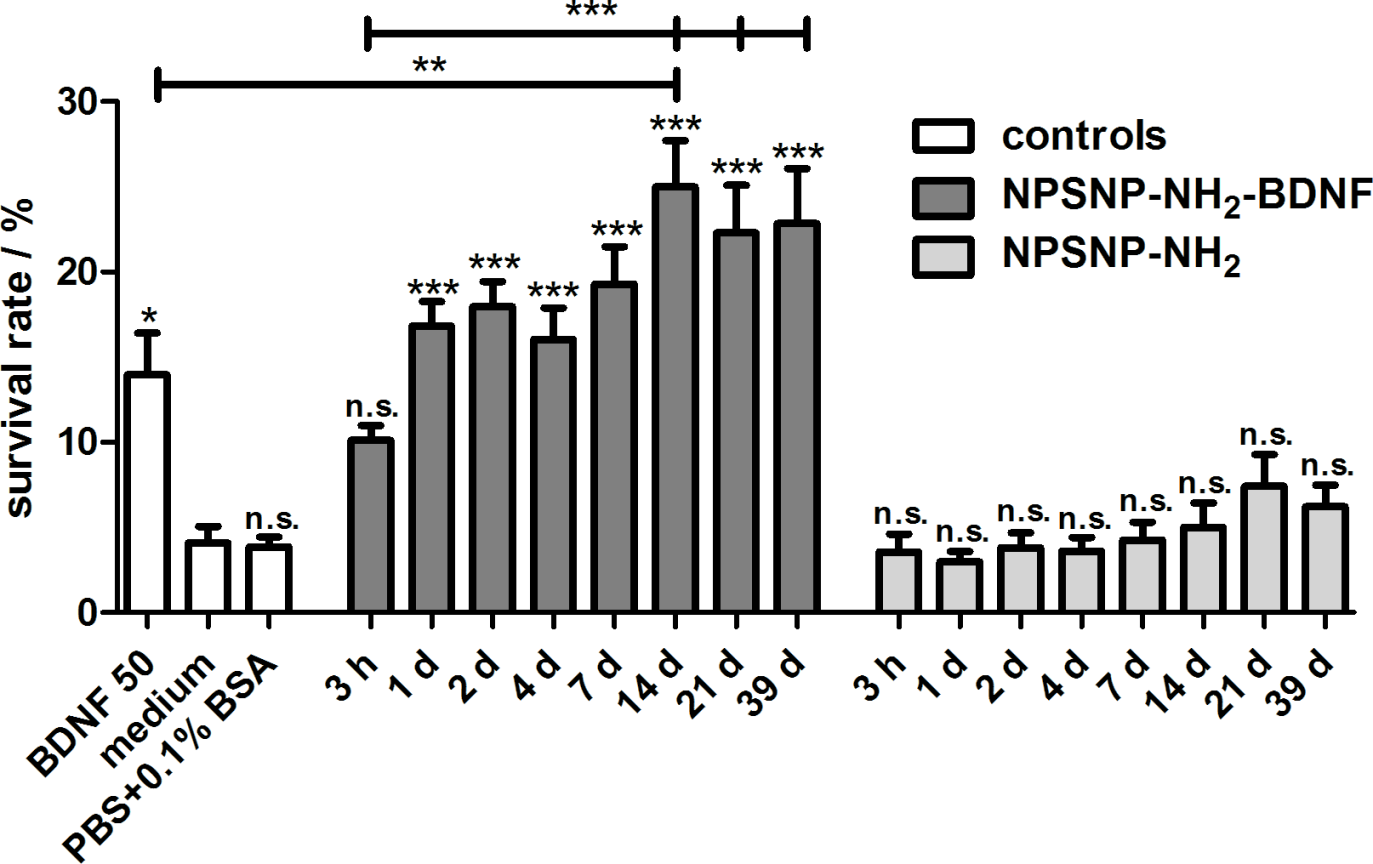
Survival rates of spiral ganglion neurons after a cultivation of two days. Cells were cultivated in presence of the supernatants from the release experiments of BDNF-loaded amino-modified NPSNPs (NPSNP-NH_2_-BDNF) or of amino-modified NPSNPs (NPSNP-NH_2_) as control experiment. Values are given as mean ± standard error of the mean (*N* = 3, *n* = 3). Statistical assessment was performed using one-way ANOVA with Bonferroni´s multiple comparison test (n.s. = not significant, *p<0.05; **p<0.01; ***p<0.001). Asterisks over the bars indicate the significance of the survival rates of different conditions compared to the medium control (serum-free SGC medium). Asterisks between two bars indicate the significance between the different conditions.

The determined survival rates of SGNs (Fig 11) confirm the results observed under the microscope. An increased survival rate of SGNs cultivated in supernatants collected from the release experiments of NPSNP-NH_2_-BDNF was observed in all supernatants when compared to the medium control. Except for the supernatant obtained after 3 h, the survival rates increased significantly (p<0.001). The comparison of the supernatants from the release experiments showed that the survival rates of the supernatants obtained from NPSNP-NH_2_-BDNF are higher than the survival rates of the control supernatants from NPSNP-NH_2_. All supernatants, except for the one collected after 3 h, led to a survival rate around 20%. The low survival rate observed after treatment with the supernatant obtained after 3 h corroborate the results obtained from the release experiments, where only a small amount of BDNF had been released after 3 h. In comparison, all supernatants of the control release experiment led to low survival rates of SGNs, which are comparable with the negative control (culture medium) and with the additional control (PBS+0.1% BSA).

**Fig 12:**
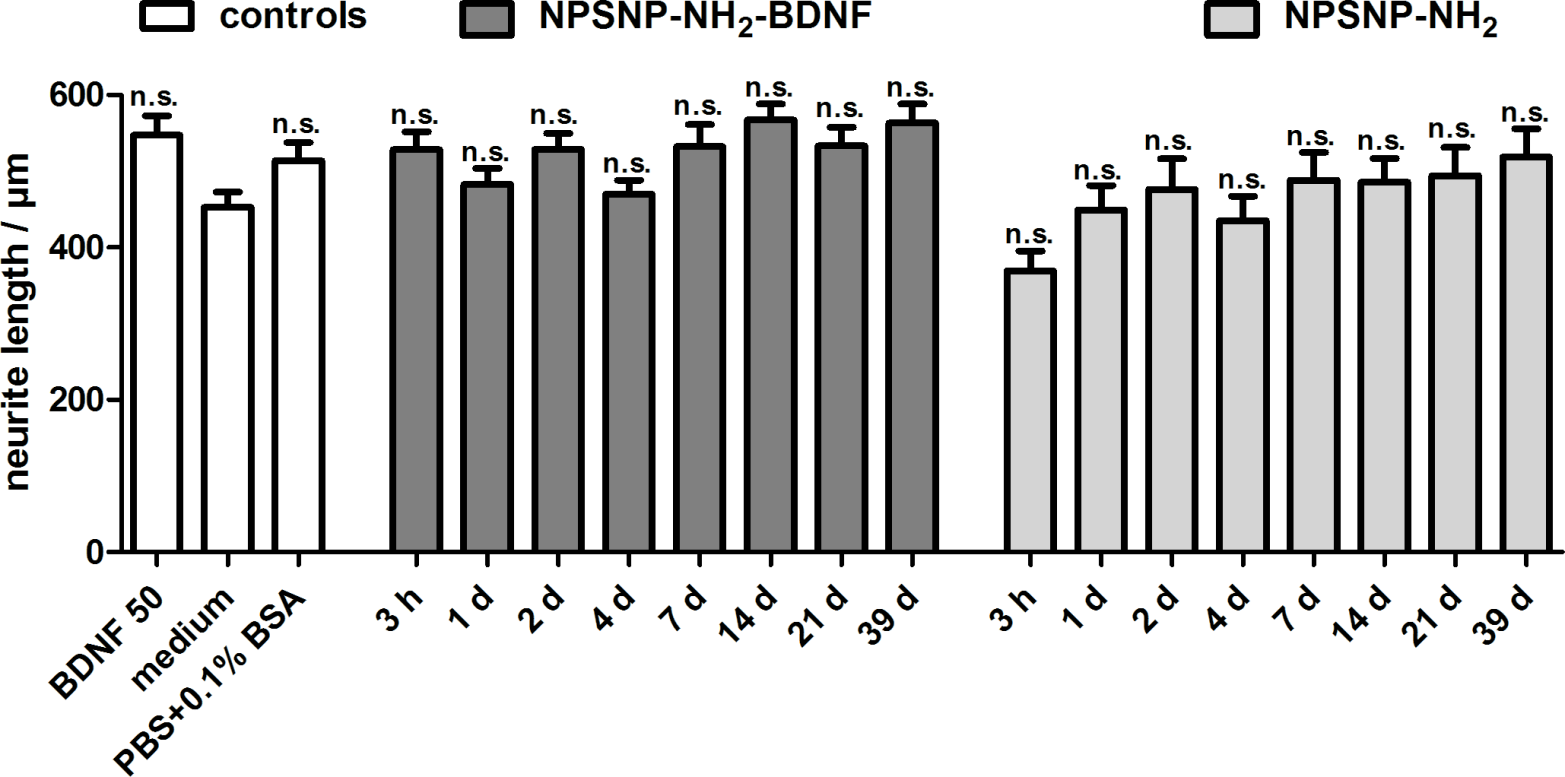
Neurite length of SGNs cultivated in supernatants of BDNF-loaded (NPSNP-NH_2_-BDNF) and BDNF-free NPSNPs (NPSNP-NH_2_). Values are given as mean ± standard error of the mean (*N* = 3, *n* = 3). Statistical assessment was performed using one-way ANOVA with Bonferroni´s multiple comparison test (n.s. = not significant, *p<0.05; **p<0.01; ***p<0.001). Asterisks over the bars indicate the significance of the survival rates of the different conditions compared to the medium control (serum-free SGC medium).

For the quantitative evaluation of a possible neuroregenerative effect of the tested supernatants on SGNs, the neurite lengths of the survived SGN were measured and are depicted in Fig 12. Among the different control groups in the experiments, the longest neurites were detected in the control with 50 ng mL^-1^ BDNF. Cultivation with supernatants of BDNF-loaded NPSNPs (ranging from 467 ± 19 μm to 567 ± 21 μm) resulted in slightly longer neurites than supernatants of BDNF-free NPSNPs (ranging from 369 ± 26 μm to 519 ± 37 μm). Although the effect is below significance, a slight trend can be observed, especially when the neurite lengths observed on samples taken at the same time point of release are compared. This indicates a slight neuroregenerative effect exerted by the supernatants derived from NPSNP-NH_2_-BDNF release, in addition to the strong neuroprotective effect.

Both, BDNF-loaded nanoparticles as well as the supernatants containing released BDNF obtained up to a release time of 39 days showed a positive effect on the survival of SGNs. Interestingly, BDNF delivered via NPSNPs is highly biologically active although the molecules underwent adsorption/desorption processes. Noteworthy, the effect is even higher than that of the control sample where the culture medium was supplemented with recombinant BDNF at a highly protective concentration.

Apart from *in vivo* experiments with delivery systems based on silica supraparticles described above [10,43], other studies described a similar effect of released BDNF. Tan et al. used BDNF-encapsulated poly(L-glutamic acid) (PGA) particles and showed that released BDNF prevented the apoptosis of SH-SY5Y cells *in vitro* [44]. Based on the neuroprotective effect of released BDNF, we presume that our nanoparticle formulation can be applied in electrode coatings to protect SGNs and to increase their survival. Based on our results, a direct contact of the nanoparticles and the neurons is not necessary. In order to equip neuronal prostheses with our nanoparticles, the incorporation in biodegradable polymeric coatings presents an interesting strategy [60]. Chikar et al. [38] and Kim et al. [75] showed that coating of electrode arrays with alginate hydrogel loaded with poly(lactic-co-glycolic acid) (PLGA) particles is a feasible approach. Loading of BDNF into the PLGA particles was demonstrated [38]. However, only a minimal release of BDNF could be observed in this case. The combination of our functioning nanoparticle-based delivery system with implant coatings has clear potential and will be developed in further investigations with regard to clinical application.

## Conclusions

In this study, we have presented the basis for an effective delivery system for the neurotrophin BDNF based on NPSNPs. After modification of the nanoparticles with amino groups, biologically relevant amounts of BDNF can be loaded to the silica surface and can be successfully released. Beside the general cytocompatibility with fibroblasts, for the amino-modified nanoparticles also the cytocompatibility for more sensitive cells, the SGNs, was demonstrated. Due to the fact that a sustained BDNF release from amino-modified nanoparticles was obtained over 80 days and the release seems not to have ceased, a long-term neurotrophic supply to prevent the degeneration of SGNs is possible. Furthermore, in the *in vitro* experiments neuroprotective effects of BDNF-loaded nanoparticles and of BDNF amounts released during a long time–up to 39 days–were proven. Especially, the neuroprotective effect of released BDNF amounts is quite interesting, since this indicates that a direct contact between the nanoparticles and the neurons is not necessary. According to this and to the long-term delivery characteristics of BDNF over 80 days, the amino-modified NPSNPs have clearly demonstrated their applicability for cochlear implant-associated growth factor delivery systems.

In general, apart from *in vitro* experiments [52,53], several *in vivo* experiments have shown that silica nanoparticles are biocompatible and biodegradable [69,70,76]. Up to now, however, they have not been registered for clinical use. Nevertheless, the first in-human study of these particles is very promising for the future [54]. In parallel to our study, other current research confirms the efficacy of silica-based carriers, because BDNF-delivering supraparticles (≈500 μm) based on this material improved the SGN survival *in vivo* [10]. Moreover, when used as delivery system in the inner ear, silica nanoparticles did not lead to hearing impairment [55]. Wise et al. assumed that the dissolved silica can be removed via the cochlear vasculature [10].

The large pore volume of our NPSNPs which is not used for the immobilization of BDNF can be applied to incorporate other small molecules like rolipram or creatine. Several studies have demonstrated a neuroprotective effect of rolipram [77,78] and that creatine can support and promote the neuronal differentiation [79]. Especially, a co-application of rolipram or creatine with BDNF has been shown to enhance the survival-promoting effect of BDNF [3,79]. Future studies of NPSNPs with rolipram or creatine incorporated into the pores and BDNF immobilized on the outer surface could potentially improve the efficacy of our delivery system. A next step would be the combination of our BDNF-delivering NPSNPs with an electrode coating, e.g. with a polymer, or the attachment of such NPSNPs to the silicone surface in order to construct a cochlear implant-based growth factor delivery system. Nanoparticles like the ones described here are easy to handle in the preparation of corresponding nanocomposites. Finally, such systems are also attractive for other stimulating and receiving neuronal electrodes.

## Supporting information

S1 Fig Particle size distribution of the unmodified (left) and amino-modified nanoparticles (right) determined by DLS. In addition to the three individual measurements of each nanoparticle type, the means and the corresponding standard deviations are shown.
(TIF)

S2 Fig Nitrogen sorption isotherms of unmodified and amino-modified NPSNPs at 77 K. (TIF)

S3 Fig TEM images of the unmodified (NPSNP) and amino-modified nanoparticles (NPSNP-NH_2_) after different treatments. Particles are shown directly after their synthesis (top figures), after an incubation in PBS (0.1% BSA) solution (simulating the loading procedure) and after 7 d and 14 d of a simulated release. The incubation was carried out without (control) or with BDNF (-BDNF). For the TEM preparation the freshly prepared nanoparticles were dispersed in ethanol and all other samples in water.
(TIF)
