# Supplementary figures and images for: Long-term delivery of brain-derived neurotrophic factor (BDNF) from nanoporous silica nanoparticles improves the survival of spiral ganglion neurons in vitro
Source: PLoS One. 2018 Mar 27;13(3):e0194778. doi: 10.1371/journal.pone.0194778 (PMC5870973; doi:10.1371/journal.pone.0194778)

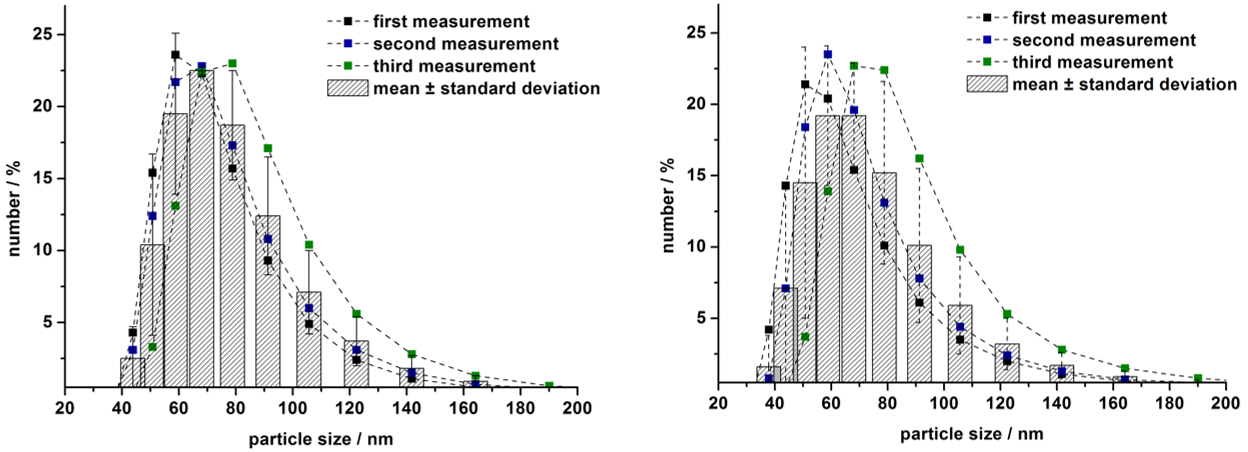

Supplement: S1 Fig — In addition to the three individual measurements of each nanoparticle type, the means and the corresponding standard deviations are shown. (TIF) [file pone.0194778.s001.tif]

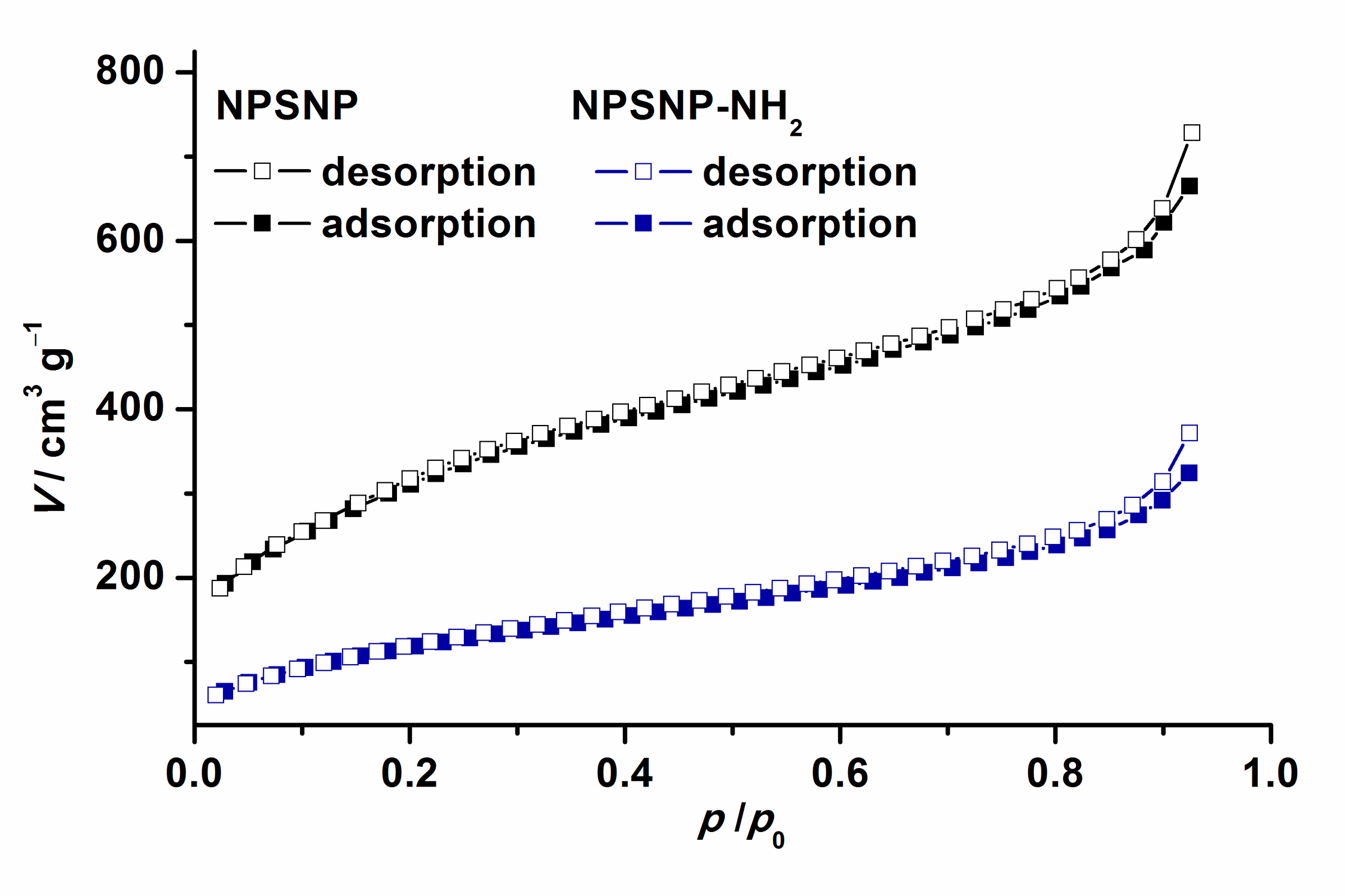

Supplement: S2 Fig — (TIF) [file pone.0194778.s002.tif]

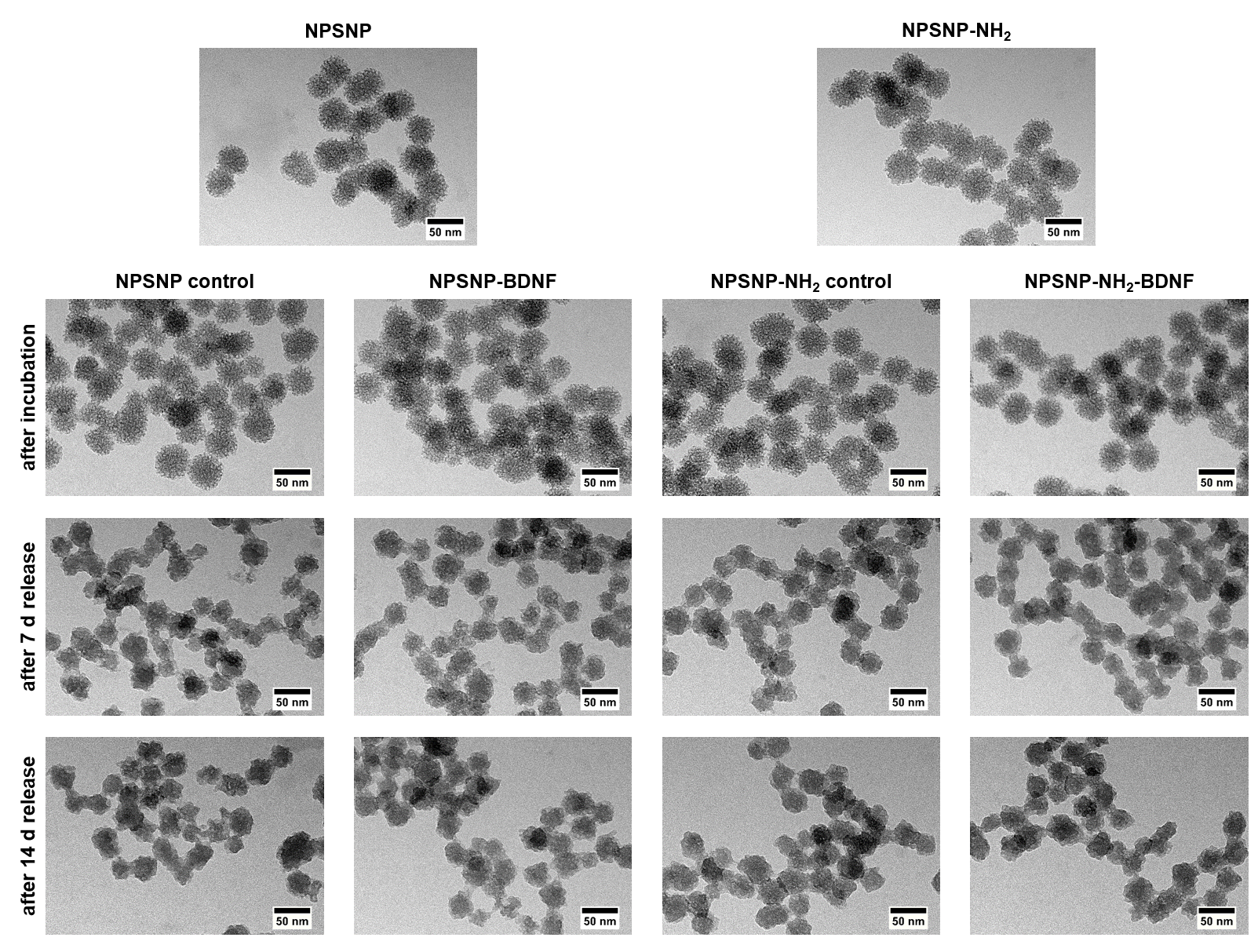

Supplement: S3 Fig — Particles are shown directly after their synthesis (top figures), after an incubation in PBS (0.1% BSA) solution (simulating the loading procedure) and after 7 d and 14 d of a simulated release. The incubation was carried out without (control) or with BDNF (-BDNF). For the TEM preparation the freshly prepared nanoparticles were dispersed in ethanol and all other samples in water. (TIF) [file pone.0194778.s003.tif]
